# The cell origin of reactive oxygen species and its implication for evolutionary trade-offs

**DOI:** 10.1098/rsob.240312

**Published:** 2025-04-16

**Authors:** Maïly Kervella, Fabrice Bertile, Frédéric Bouillaud, François Criscuolo

**Affiliations:** ^1^ EPE, IPHC, Strasbourg, Grand Est, France; ^2^ Institut Cochin Département EMD, Paris, Île-de-France, France

**Keywords:** ageing theory, bioenergetics, longevity, oxidative metabolism, oxidative stress

## Abstract

The allocation of resources in animals is shaped by adaptive trade-offs aimed at maximizing fitness. At the heart of these trade-offs, lies metabolism and the conversion of food resources into energy, a process mostly occurring in mitochondria. Yet, the conversion of nutrients to utilizable energy molecules (adenosine triphosphate) inevitably leads to the by-production of reactive oxygen species (ROS) that may cause damage to important biomolecules such as proteins or lipids. The ‘ROS theory of ageing’ has thus proposed that the relationship between lifespan and metabolic rate may be mediated by ROS production. However, the relationship is not as straightforward as it may seem: not only are mitochondrial ROS crucial for various cellular functions, but mitochondria are also actually equipped with antioxidant systems, and many extra-mitochondrial sources also produce ROS. In this review, we discuss how viewing the mitochondrion as a regulator of cellular oxidative homeostasis, not merely a ROS producer, may provide new insights into the role of oxidative stress in the reproduction–survival trade-off. We suggest several avenues to test how mitochondrial oxidative buffering capacity might complement current bioenergetic and evolutionary studies.

**Figure:**
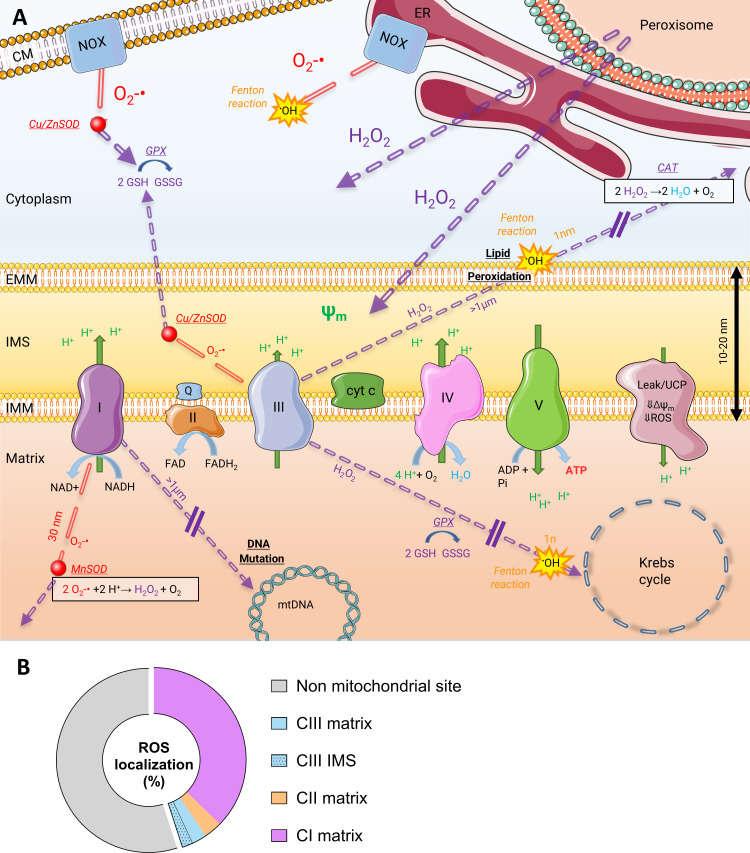

## 1. Introduction: fitness and the reactive oxygen species theory of ageing

Animals depend on energy from the environment to sustain vital functions and reproduce. The allocation of acquired energy to somatic and reproductive processes is subject to trade-offs. Thus, if immediate survival is compromised, resources are reallocated towards short-term survival at the expense of long-term processes (growth, maintenance, reproduction). These trade-offs shape life-history strategies selected in a way that maximizes individual fitness in given environments.

Food is used either to provide energy that is used to fuel metabolic processes or to increase individual biomass. Consequently, the total metabolic rate—the rate of energy expenditure—is typically higher than the energy cost of basal maintenance, as additional energy is required for cellular processes (including biomass increase). Here, we define energy metabolism as oxidative metabolism, broadly understood as the consumption of O_2_ and the release of CO_2_. Later, we will use the term respiration in a more restrictive sense.

Faster growth and higher reproductive rates are often associated with higher metabolic rates and oxidative costs, which can contribute to shorter lifespans, thereby characterizing the pace-of-life continuum [1–3]. A mechanistic explanation would be based on the proportionality between mitochondrial respiration and the release of reactive oxygen species (ROS). Within this (still disputable) [4] free radical theoretical context, the inevitable by-product of ROS during mitochondrial respiration is viewed as the principal determinant of ageing since ROS can lead to damages at the molecular level with consequently deleterious effects across higher biological scales (from cellular and tissue to individual and consequences at the population level) [2]. Note that mitochondria possess their own genome, and interactions with nuclear genes determine mitochondrial functions, including oxidative stress response (i.e. cellular processes that detect, neutralize and repair the damage caused by ROS). This relationship between two genomes can therefore result in different phenotypes and life history traits.

The hypothesis that the rate of ROS production is related to the rate of O_2_ consumption provided a *prima facie* explanation for why fast-living animals were often short-lived: they chronically suffered from higher levels of oxidative stress [5]. Yet, limitations to this model rapidly became apparent [6]. Evidence started to accumulate over subsequent years against the simple explanatory diptych ‘high metabolism/high ROS’ for different ageing rates or longevities [7,8]. The observation that individuals with a high metabolism may still live longer than conspecifics with lower metabolic rates [9] triggered a deeper interest in specific mitochondrial adaptations [10].

### 1.1. Energy metabolism, mitochondria and reactive oxygen species formation

Hydrolysis of the third phosphate bond of adenosine triphosphate (ATP) powers most energy-demanding cellular processes, leading to the formation of adenosine diphosphate (ADP). The phosphorylation of ADP into ATP involves gas exchanges as written below. First, oxidation of carbon-containing molecules (such as sugars, lipids and proteins) releases electrons (*e*^−^) and CO_2_ (during reduction of pyruvate into acetyl-CoA and Krebs cycle steps). This carbon oxidation is accompanied by substrate-level phosphorylation in the enzymatic steps of glycolysis and the Krebs cycle, generating some ATP (four per glucose). However, the largest part of the energy released from this carbon oxidation resides in the electrons extracted and loaded onto redox shuttles (coenzymes) such as NAD^+^/NADP^+^, which are reduced to NADH/NADPH, or FAD/FMN reduced to FADH_2_/FMNH_2_. This process implies the transfer of two electrons and one or two protons.

O_2_ is then used in oxidative phosphorylation (OxPhos). OxPhos is the oxidation of the reduced coenzymes by the mitochondrial respiratory chain also named the electron transport chain (ETC). The ETC transfers electrons to the final acceptor O_2_ and releases water (this is respiration *stricto sensu*) while extruding protons in the intermembrane space of the mitochondria, thereby creating a proton motive force used by ATP synthase (complex V of OxPhos). Both processes are coupled, meaning that electron transfer in the ETC is controlled/limited by the ATP formation rate through ATP synthase. OxPhos is so far the largest consumer of O_2_ in a cell and results in the formation of ATP at high yield, with approximately 30 molecules of ATP produced from the oxidation of one glucose molecule and 40 ATP produced from the oxidation of six carbon fatty acids. The complete oxidation of glucose reaches a maximum P/O ratio of 2.79, meaning that 2.79 moles of ADP are phosphorylated to ATP for every pair of electrons (2*e*^−^) flowing through the electron transfer chain to reduce one molecule of oxygen [11]. The yield is never maximal because of the variability of the coupling between substrate oxidation and ATP formation. Current values of the coupling (yield of OxPhos) range from 80% to 50–40% [12,13] and entail a range of incertitude for the proportionality between ATP turnover and O_2_ consumption. The molecular explanation is a percentage of protons that escapes the OxPhos circuit essentially through leaks that enable proton return via other pathways than through ATP synthase.

**Figure 1.**
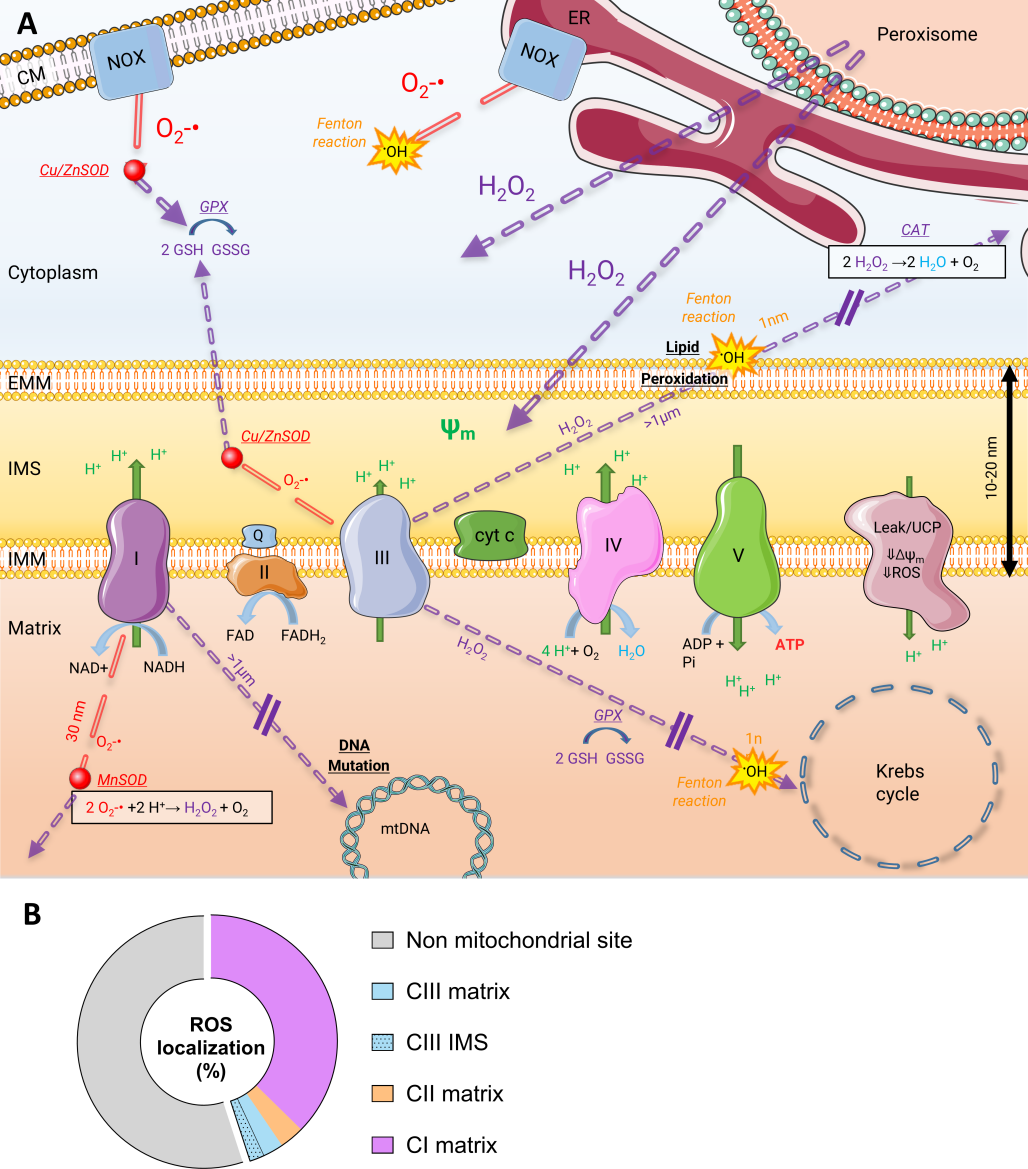
Oxygen reduction in biological systems and ROS production. (A) ETC is the main consumer of electrons. During their transport through the ETC, protons are pumped from the matrix into the intermembrane space by complexes I, III and IV. Electron transport ends with the reduction of an oxygen molecule (O_2_) into two molecules of water by complex IV (cytochrome oxidase). In the process, the couple NAD/NADH is the main electron donor associated with carbon oxidative metabolism (at complex I). H^+^ active pumping creates an electrochemical gradient, ψ_m_, that is used to convert ADP into ATP by complex V (ATP synthase). Note that the proton return to the mitochondrial matrix can also be made through basal proton leak or inducible proton leak (UCPs/ANT). These mechanisms decrease the membrane potential, which decreases mROS production. mROS are mainly produced by CI and CIII (some ROS are produced in CII, data not shown). Both complexes produce mROS in the mitochondrial matrix, but CIII produces ROS into the IMS too. Mitochondria are not the only ROS producers in cells; they are, for instance, also produced in the cytoplasm by NOX at the ER, and the CM. Electron leak leads to O_2_^−•^ when there is the reduction of a single electron, H_2_O_2_ is formed when a pair is reduced. O_2_^−•^ is very reactive and damages neighbouring biological molecules within a short time (migration distance can reach 30 nm). While H_2_O_2_ is less reactive (that is why the distance migration is higher >1 µm), it is thought to release OH° (Fenton reaction), a strongly reactive radical that attacks every close biomolecule type (DNA, RNA, proteins and lipids, distance migration 1 nm). Cells are equipped against these deleterious molecules: superoxide dismutase (MnSOD in mitochondria, Cu/Zn SOD in intermembrane space and cytoplasm) dismutases O_2_^−•^ into oxygen and H_2_O_2_, which can diffuse into the cytoplasm; catalase reduces H_2_O_2_ into water and oxygen, glutathione peroxidase into water and glutathione disulfide. Non-enzymatic scavenging systems, such as glutathione (GSH/GSSG), are also involved. (B) On a cellular level, mitochondria produce less than half ROS, with values that may vary by an order of magnitude, depending on the cell type [14,15]. Here, we schematically represent the sites where ROS are located, based on various literature data, whether focused only on mitochondria or not, to provide a general idea at the same cellular scale. The mtROS percentage is derived from data on rat liver [15], but can be halved or doubled based on other values [14]. The mROS values shown here are from Quinlan’s work [16], which concerns isolated mitochondria from skeletal muscle. The results are here reported as a percentage of the total amount of ROS. Muller’s values from mouse skeletal muscle complete the ROS location in the organelle [17]. Additional examples of the mROS/total ROS ratio can be found in [14]. CM, cell membrane; ER, endoplasmic reticulum; EMM, external mitochondrial membrane; IMS, intermembrane space; IMM, external mitochondrial membrane; ETC, electron transport chain; CAT, catalase; GPX, glutathione peroxidase; GSSG, oxidized glutathione; GSH, reduced glutathione; UCP, uncoupling protein.

Oxygen reduction for energy production in biological systems and associated ROS production are resumed in figure 1. According to the literature, 0.2−2% of the electrons in the ETC are considered to leak out of the chain (to superoxide (O_2_^−•^) formation) before reaching the complex IV reaction [18,19]. However, as shown in figure 1A, the production of ROS, such as O_2_^−•^ and hydrogen peroxide (H_2_O_2_), is not restricted to the mitochondrial ETC and could be generated by other cellular compounds (and not only mitochondrial) such as NAD(P)H oxidases. Importantly, radicals (and not necessarily oxygen radicals) are obligatory intermediates in some enzymatic reactions. Therefore, cellular ROS formation could result from reactions with a given purpose (defence against pathogens, reaction intermediates, etc.) from unexpected leakage of electrons forming ‘orphan ROS’ with increased risk of oxidative damage to cellular components.

Cell defences against ROS include scavenging radicals or repairing damaged molecules. To date, 11 mitochondrial sites have been identified to produce H_2_O_2_ and/or O_2_^−•^ *in vitro*, in isolated mammalian mitochondria [20]. However, the intensity of mitochondrial fluxes involved in energy metabolism leads (perhaps abusively) to consider mitochondria as the most important cellular ROS generator. Studies measuring the involvement of each ETC complex in ROS production showed that ROS were primarily produced mainly by the ETC complexes CI, then CIII and finally CII [21,22], even though species-, tissue- and experimental condition-dependent variations were observed. In relative terms, Quinlan *et al.* measured H_2_O_2_ production in rat skeletal muscles and found that CI was responsible for 83% of ROS generation, CIII 10% and CII 7% [16]. Complex I deficiency has been identified as the most frequently defective ETC enzyme in patients suffering from mitochondrial disorders, and studies revealed that elevated mitochondrial ROS (mtROS) production negatively correlated with residual activity of the defective complex [23,24]. In terms of topology, complexes I and II produce ROS only in the mitochondria matrix, whereas complex III produces ROS both in the matrix and intermembrane spaces (figure 1B) [17,25]. The mtROS produced in the intermembrane space theoretically have easier access to the cytosol than those produced in the matrix, as they are only separated from that compartment by the outer mitochondrial membrane. This might explain how ROS site production could play different roles in redox signalling and oxidative stress. Bleier *et al.* [25] analysed the proteome of isolated mitochondria from rat heart and showed that redox-sensitive protein targets were differently affected when complex I or complex III was used as a source of ROS, even in the matrix [25]. Still, at the level of the cell, only half of the total ROS amount has been related to mitochondria in those studies, NADPH oxidases (NOX) having been recognized as the main source of ROS in numerous cell types, including the skeletal muscle [26].

### 1.2. Mitochondrial implications in the diversity of ageing

Mitochondrial defects are one of the hallmarks of ageing and declining health [27,28]. Consequently, mitochondria attracted the interest of researchers from many biological fields, and evolutionary biology, which focuses on the understanding of the life-history trade-offs that led life forms to evolve as they are present, is not an exception. For roughly 20 years, the way ATP is produced and its concomitant oxidative cost have been studied as a key mechanism underlying the trade-off between growth, reproduction and lifespan (e.g. [29]). The efficiency of mitochondrial ATP production must have evolved under selective pressure from limited energy availability, with life-history traits continuously competing for this finite energy pool to promote individual fitness [30,31].

Investigations on the role of mitochondria in the mechanistic basis of life history trade-offs have focused on the links between ATP turnover, ROS production and the physiological and fitness consequences of the coupling level of OxPhos (i.e. thermic loss from proton leakage contributing to organismal thermogenesis [16]. In that context, researchers rapidly proposed a re-evaluation of mitochondrial uncoupling as a basis of the ‘live fast, die young’ hypothesis to explain why some fast O_2_ consumers were not always species or individuals showing shortened lifespans (see uncoupling section). However, even under the so-called *uncoupling to survive hypothesis* [32], mtROS production remains a key variable modulating animal life history.

### 1.3. The alternative view of the mitochondrial role in the regulation of oxidative stress

Several reasons have led to reconsider mtROS production as a main issue for organisms. First, mtROS are not only harmful molecules but also fulfil a signalling role in many intracellular pathways [33,34], suggesting that their production is actually necessary. Researchers thus began to question the role of mitochondria as a main source of ROS production within the cell, based on the observations that ROS production by mitochondria is not high *in vivo* [35] or when measured under physiological conditions *in vitro* [36]. However, studies focusing on ROS production mechanisms *in vitro* typically worked at O_2_ saturation levels (i.e. 100%, roughly 200 µM at 37°C) not representative of mammalian physiological O_2_ saturation levels, at least 10 times lower in the extracellular fluid (less than 20 µM) [37] and presumably much lower at the sub-level of mitochondria. In addition, mitochondria share the ability to produce ROS with many other cell sites, such as the peroxisome, endoplasmic reticulum and plasma membrane [14]. Non-mitochondrial ROS may, in fact, account for over 50% of the cell’s total ROS production (figure 1B). Several enzymes, including those of the NADPH oxidase family, catalyse the production of O_2_^−•^ and H_2_O_2_ while oxidizing NADPH to NADP [38]. Such enzymes may actually produce more ROS than mitochondria [14,39]. Additionally, non-mitochondrial ROS-producing enzymatic sites have been further identified [40,41].

The intricate cross-talk that exists between all cellular (including mitochondrial [42,43]) ROS producers results in synergistic and antagonistic interactions that add complexity to our understanding of how the oxidative cell status is regulated [39] and how selection pressures related to oxidative stress may drive the evolution of heritable traits [30]. Finally, mitochondria are well-equipped with antioxidant systems, from superoxide dismutase, thioredoxins and enzymes of the glutathione system to catalase, which collectively provide mitochondria with a theoretically high ROS-buffering capacity [44]. There are multiple examples showing that mitochondrial antioxidant activity can rapidly quench oxidative threats [45–47] and that mtROS consumption may often outperform mtROS production [44], with ROS scavenging capacity of brain mitochondria reaching, for instance, 100 times the rate of ROS generation [48].

This review will not go further in describing the rationales of the free radical theory of ageing [3], the challenges faced by this theory [4,49,50] or non-mitochondrial ROS cell systems [51] and their interconnection with the mitochondria [43,52]. We aim to add a complementary perspective to the general idea that the way mitochondria produce ATP and ROS primarily defines the trade-off between reproduction or longevity at the individual level [53,54]. We shortly present ideas for future studies to test how the alternative view of mitochondria as a component of cellular homeostasis in terms of energy and antioxidant activity adds to our understanding of trait evolution. We present how these metabolic regulatory properties of mitochondria may counterbalance the non-mitochondrial ROS production and modulate the reproductive trade-offs.

## 2. Alternative mitochondrial roles in the context of cost of reproduction

**Figure 2.**
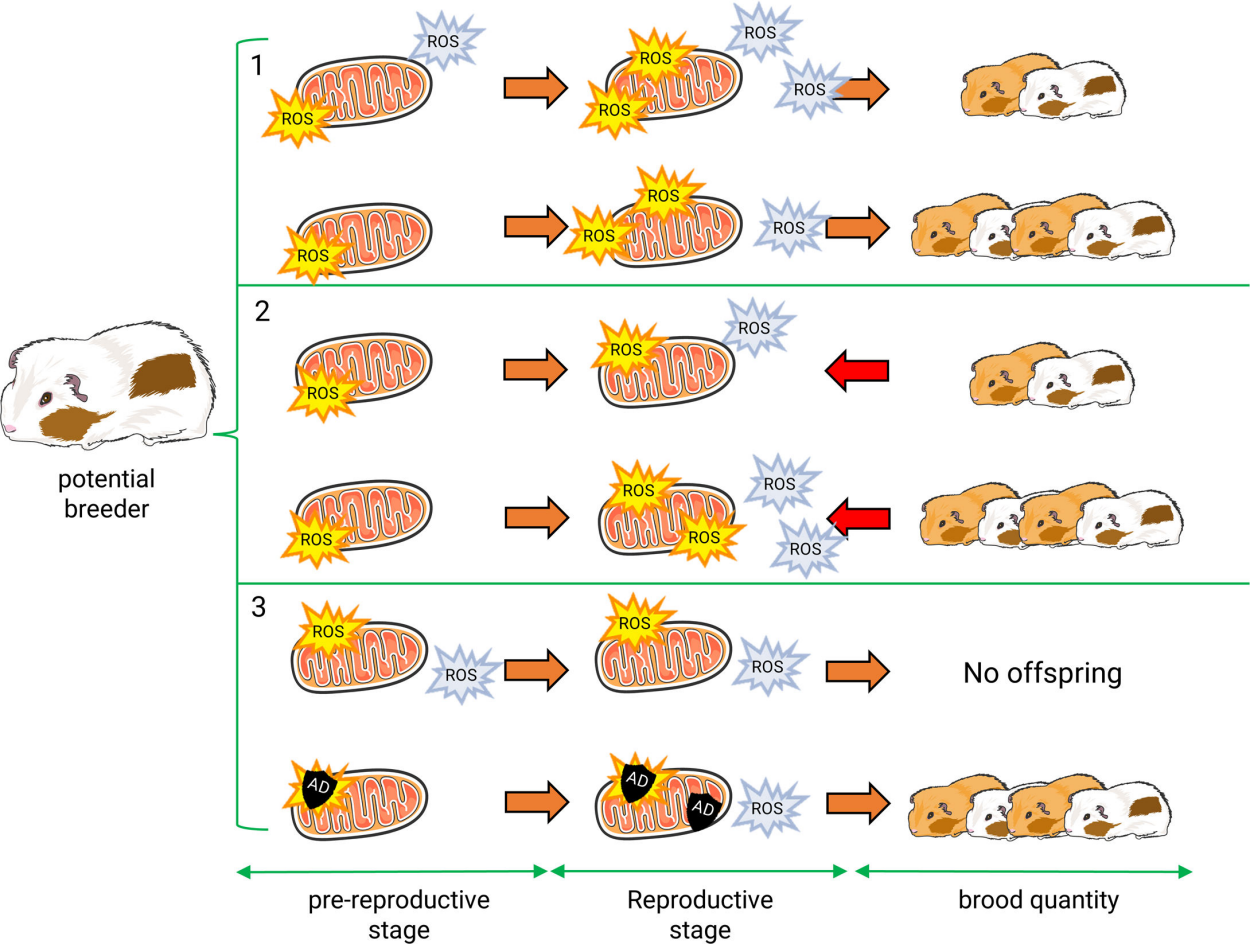
Mitochondrial involvement in reproductive effort. (1) *Oxidative stress constrains* reproduction and limits parental investment, according to the constraint hypothesis. Thus, females with lower levels of oxidative stress during the pre-reproductive stage can invest more into reproduction, the hypothesis assuming an increase in ROS production due to the reproductive investment and a lack of antioxidant defences due to the choice in energy allocation. ROS are produced by mitochondria, but also elsewhere. (2) Following the *cost hypothesis*, oxidative stress increases with maternal reproductive investment/metabolism, with antioxidant capacities being not sufficient. (3) A pre-reproductive increase in antioxidant protection and/or decrease in ROS production leads to the *oxidative shield hypothesis*, in which we propose a key role of mitochondria as a ROS quencher. By this, mothers preserve offspring from maternal oxidative stress *in utero* and during lactation stages. Here, explosion icons portray ROS production, with yellow for mtROS and blue for other production sites such as NOX. Black shields are for mitochondrial antioxidant capacities (AD for antioxidant defences). During the pre-reproductive stage, all mitochondria produce antioxidants. With a view for simplification, we only showed them for the oxidative shield hypothesis, where they are more produced.

Building on the physiological and energetic foundations of life-history traits [55–58], studies on how oxidative stress influences trade-offs between growth, reproduction and lifespan in animals have flourished over the past couple of decades (e.g. [59–61]). These studies introduced the imbalance between ROS production due to high levels of energy expenditure [62] and antioxidant defences as a plausible mediator for the cost of reproduction. However, the hypothesis that an increased accumulation of oxidative damages due to reproductive effort caused somatic costs to the organism (the ‘oxidative cost’ hypothesis) rapidly faced inconsistencies (e.g. [63]), the most important being that breeders often suffered from less oxidative stress than non-breeders [64,65]. This led to the ‘oxidative shielding hypothesis’ (OSH) [66] (figure 2) proposing that the oxidative costs of reproduction may be masked if adult breeders anticipated the increase in ROS production expected during reproduction by pre-emptively increasing their antioxidant defences to shield their developing offspring from harmful molecules [66,67]. Alternatively, the oxidative status of breeders before entering reproduction may also limit reproductive investments (i.e. oxidative constraint hypothesis [68], contributing to explaining lower oxidative stress in breeders if oxidative status serves as an endogenous signal to optimize the trade-off between current and future reproduction [68,69]. Measuring antioxidant levels and oxidative damage before, during and after reproduction in both breeders and non-breeders [67,70], or after manipulating oxidative status (e.g. through energy/resource constraints [70] or pro-oxidant diets) or reproductive cost [71], should clarify whether the reduction in oxidative damage in breeders results from a proactive protective mechanism (OSH) or from a pre-existing constraint limiting reproductive effort, such as the capacity to dissipate metabolic heat [71].

In the context of OSH, the antioxidant role of mitochondria may not only be central, limiting ROS-induced damage, but also freeing up energy for general body maintenance (e.g. protein turnover), with long-term benefits for breeders. In fact, the distinction between oxidative damage that impacts the functionality of biomolecules and ROS generation may deserve attention. The extent of oxidative damage is the result of the imbalance between the generation of the damage (ROS action) and repair. Repair implies the use of energy, especially if replacement of damaged proteins is required (four ATP *per* amino acid added to a polypeptide chain). Protein synthesis depends on energy availability and is more strongly downregulated when energy availability is low than are mechanisms ensuring ionic balance [72–74]. The rate of protein synthesis has thus been proposed as a proxy for cellular metabolic status (SCENITH approach [75]). An increase in oxidative damage could result from a reduction in the mitochondrial antioxidant capacity, forcing cells to redirect energy towards repairing damaged biomolecules. In this context, oxidative damage may reflect an overall compromised antioxidant system (and not just a transient increase in mitochondrial ROS production), leading to a long-term deleterious loop. Indeed, the additional energy required for repair diverts resources away from general maintenance, resulting in an imbalance that compounds the initial problem. Such a metabolic compromise may have broader mid- to long-term consequences beyond the oxidative damage itself, potentially affecting overall cellular homeostasis and fitness.

Alternatively, investments into somatic repair may be less selected than investments into reproduction for species with a fast pace of life [76]. To avoid the self-reinforcing cycle of continuous biomolecular repair, which diverts energy from basal turnover of biomolecules that maintain homeostasis over the long term, long-lived species should ensure that oxidative damage is minimized by strict control at the source (i.e. buffering of non-mitochondrial ROS production) and not constantly repaired. Consequently, along the pace of life continuum (from fast to slow living species), the antioxidant impact of mitochondria may be of increasing importance as mtROS production rate is efficiently buffered. This is particularly relevant because damage from even low levels of ROS can accumulate over a longer lifespan. Thus, while fast-living species often prioritize investment in growth/reproduction, leading to a reduced focus on the prevention or repair of molecular damage [77–79], an early buffering of oxidative stress (i.e. by mitochondria at the cell level) remains crucial for slow-living species. The latter must preserve cellular integrity for extended periods, and the inadequate management of ROS production leading to oxidative damage could jeopardize survival and future reproductive success [80,81]. Consequently, species that favour rapid reproduction may exhibit less sophisticated mechanisms for mitigating oxidative stress, while those that invest more in long-term survival might benefit from better regulation, including mechanisms like mitohormesis (see below).

In summary, studies considering the fine-tuning of mitochondrial ATP versus ROS production and the mobilization of cellular antioxidants in the framework of life-history trade-offs have mostly focused on testing the cost of reproduction [61]. However, there are several additional arguments to propose a more integrated and proactive role for mitochondria in the regulation of oxidative stress.

### 2.1. Mitochondrial oxidative metabolism controls adenosine triphosphate and redox status

The primary role of mitochondria is to produce ATP. Based on the observation that a peak in metabolic activity such as sustained endurance is not necessarily followed by an immediate rise in oxidative damage (e.g. [82]), we hypothesize that, in most cases, the ROS release accompanying reproduction, which also increases metabolic activity, is not of mitochondrial origin. Oxidative stress may not be an obligatory issue of high mitochondrial respiration. Rather, mitochondrial ATP production should adjust to the increased energy demand triggered by breeding by ensuring the tight regulation of ROS through mitochondrial antioxidant function and ROS-scavenging mechanisms. Under this hypothesis, mitochondrial ROS production being under control, non-mitochondrial ROS should be the target of concern when considering reproduction-associated ROS release. This balance between energy production and ROS management is critical to interrupt the oxidative damage chain at an early stage, which could otherwise compromise reproductive success and survival. This was observed in house mice (*Mus musculus*), in which a comparison of mitochondrial efficiency (measured as the respiratory control ratio [83]) was performed in breeders and non-breeders, highlighting no differences between the two groups after the breeding period despite a slight increase in cell membrane oxidative damage [84] (see below for an alternative explanation of this oxidative effect). Although maintaining an adequate level of ATP production is essential, the simultaneous control of mitochondrial and non-mitochondrial ROS is then equally vital. This suggests that both energy production and oxidative stress regulation are tightly linked, with selective pressures acting on both processes to ensure optimal fitness outcomes. The maintenance of an adequate level of mitochondrial respiration rate and/or of coupling of respiration with ADP phosphorylation has previously been proposed as essential factors modulating trade-offs [85]. However, it is important to note that while maintaining ATP production is indeed a high-priority target of natural selection, this does not mean that early cellular ROS buffering is of lesser importance.

### 2.2. Mitochondrial oxidative metabolism controls the production of cellular energy intermediates

A second, and key, regulatory property of mitochondrial OxPhos lies in its ability to oxidize reduced cofactors like NADH/NADPH and others (FADH_2_, FMNH_2_). In doing so, the rate of mitochondrial respiration regulates the redox state of the cell and indirectly controls a large panel of intracellular pathways of key importance to the fitness of individuals [33,34]. Mitochondrial coupling has the disadvantage of linking redox balance and ATP turnover. This prevents any fine adjustment of the redox status of the cell independently of ATP demand. To increase flexibility, pathways exist to re-oxidize reduced coenzymes independently of ATP generation, such as non-proton pumping respiratory enzymes (essentially in plants) or uncoupling proteins [10,86]. The flexibility to adjust mitochondrial functioning has provided benefits in terms of mitigating oxidative stress (e.g. immune oxidative costs) and has allowed the exaptation of UCP1 as a promoter of non-shivering thermogenesis [87–90], particularly in small mammals [86,91]. In these species, heat stress has been proposed as a physiological constraint limiting the increase in metabolism and breeding success (heat dissipation limit theory) [92,93]. Therefore, being able to transiently downregulate mitochondrial uncoupling status and its induced thermogenesis effect, as well as the consequent waste of energy into heat, may help to finely adjust ATP production to reproductive demands [94,95]. Hence, brown adipose tissue atrophies, mitochondrial content and gene expression of UCP1 are lowered in breeding females compared with non-breeding at the same temperature, while lactation is already concomitant with an increase in body temperature *per se* [94]. Such a suppression of thermogenesis in BAT has been attributed to a decrease in the sympathetic nervous system activity. However, losing these ‘wasting proton pathways’ (i.e. UCPs) may compromise the ability of mitochondria to re-oxidize respiratory chain cofactors independently of ATP demand (i.e. flexibility). NADH and other cofactors are indeed involved in processes such as cellular antioxidant capacity, cellular stress response and healthy ageing [33,34,96,97], which are all of importance for the future fitness prospect of breeders. Such inflexibility in mitochondrial functioning may lead to the progressive development of metabolic inflexibility, which is one of the hallmarks of ageing [98].

### 2.3. Antioxidant capacity: an overlooked role for mitochondria?

Mowry *et al*. [84] showed that breeding mice present increased levels of mitochondrial enzymatic antioxidants (see also [63] for a similar effect on constitutive antioxidants in reproductive birds). In addition, oxidative damages in cell membranes were marginally increased in breeders. The observations that mitochondrial integrity and efficiency are preserved and that mitochondria exhibit higher antioxidant capacity than other intracellular sites subject to oxidative damage after any energy-costly activities (including protected nuclear material such as telomeres) support the idea that if ROS production increases following high-expenditure activities, this should be primarily attributed to non-mitochondrial cellular production sites (e.g. intramembrane NOX enzymes) [44,99]. Mitochondrial antioxidant capacity is of prime importance in regulating the oxidative status of the whole cell through its ability to buffer cytosolic ROS [48]. Whereas mitochondria inevitably produce ROS, we propose that mitochondrial and non-mitochondrial ROS may trigger distinct mitochondrial responses.

Mitochondrial hormesis (or mitohormesis, reviewed in [100]) describes how mitochondrial performance follows an inverted U-curve in response to increased levels of ROS. While the rate of mtROS production is generally lower compared with other intracellular sources [14,39,99], a slight or moderate increase in mtROS levels (i.e. remaining below a harmful overexposure [101,102]), particularly during periods of metabolic shifts like reproduction, may serve as an adjustment variable for both mitochondrial and cellular functions: at these low levels, mtROS may be important to trigger early cellular signalling of adaptive responses, including interaction with NF-KB, MAPK and Ca^²+^, which are also important in the regulation of the ageing processes [103]. In other words, at low concentrations, mtROS can initiate protective mechanisms that enhance the cell’s antioxidant defences. This helps prepare countermeasures for an anticipated increase in ROS production from non-mitochondrial sources, thereby maintaining oxidative homeostasis. At this point, it is crucial to emphasize that the overall ROS levels in the cell are influenced not only by their production rates but also by their effective elimination. High mitochondrial antioxidant capacity, enhanced via mitohormesis during breeding, likely reflects an efficient elimination of both increasing mitochondrial and non-mitochondrial ROS, preventing oxidative damage. This dynamic balance reflects the long co-evolutionary history between mitochondrial and nuclear genomes, which has fine-tuned these adaptive mechanisms over evolutionary time [104,105].

When cellular ROS levels are high, mitochondria may function as an overall ROS quencher for the entire cell, neutralizing both mitochondrial ROS and non-mitochondrial ROS (H_2_O_2_) that diffuse into the organelle [14]. In fact, superoxide and/or H_2_O_2_ produced by non-mitochondrial sites may enter the ETC and oxidize cytochrome *c* and complex IV, thereby participating in ATP production [106,107]. If mtROS levels are high, this is likely the reflection of a suboptimal mitochondrial state not compatible with cell survival in the short term. mtROS may then fulfil adaptive roles in two different situations (at low rates by promoting adaptive responses or at high rates by promoting apoptosis of cells with malfunctioning mitochondria). This likely takes place independently of the level of O_2_ consumption. Then the implication of mitochondria on individual fitness is not simply linked to an increase in oxidative stress at the level of the whole organism, as initially expected in breeders [66,108,109]. It is nevertheless important to note that antioxidant systems within the cytosol are also of importance in highly energy-demanding situations (e.g. [110]) since not all the cytosolic ROS produced enter the mitochondria and are neutralized by mitochondrial antioxidant systems. Still, the activity of these antioxidant systems is subject to debate (e.g. [111]) and as they are using energy intermediates (e.g. NADPH), they may also be modulated by mitochondrial flexibility (see above).

### 2.4. Cell antioxidant capacity and non-mitochondrial reactive oxygen species: variance, heritability and fitness effects

Antioxidant defences and mitochondrial adjustments to ROS levels must nonetheless meet evolutionary prerequisites to play a significant role in maintaining trade-offs among life-history traits.

For mitochondrial regulation to play a role in life-history trade-offs, mitochondrial traits should respond to selection. This entails that inter-individual variability in antioxidant mitochondrial capacity and in the rate of production of non-mitochondrial enzymes should exist for selection to act upon. Several lines of evidence suggest this is the case. Polymorphisms in genes coding for glutathione-S transferase, catalase or mitochondrial superoxide dismutase (MnSOD) have been previously characterized [39,52,112]. Incidentally, the MnSOD is the only superoxide scavenger present in the organelle. Similarly, non-mitochondrial ROS producers like the enzymes of the NOX family present characteristics that make them susceptible targets of selection. They are present in a large number of cell types and located at different cellular sites [52], likely including mitochondria [39]. This systemic expression gives them the ability to regulate many physiological processes, either via direct damages induced by the ROS they produce (e.g. immunity [113] or indirectly via the impact of ROS on cell signalling [51]; see below). Importantly, inter-individual variations in NOX activity have been recorded in humans due to polymorphism in particular subunits or regulator gene sequences (reviewed in [39]). Such inter-individual variation may reflect inherited biological characteristics within families, which remains to be tested.

How mitochondrial (of main maternal inheritance in sexual reproducers, but see [114]) and nuclear genomes interact to define the heritability of mitochondrial function is a key question already tackled elsewhere [31,115,116]. In addition to the existing variance in the ETC functioning, additional mitochondrial regulatory processes may explain individual variance in mitochondrial efficiency (e.g. hydrogen sulfide regulation [117]).

To be under selection, the above-mentioned mitochondrial and non-mitochondrial mechanisms must modulate fitness *via* the promotion of higher reproductive success or longer lifespan in individuals. One of the ‘Oxidative stress theory’ expectations is that lower rates of mtROS production are associated with longer longevities (e.g. like in birds [118–120]). However, several studies questioned this affirmation by showing that the rate of ROS consumption by mitochondria (i.e. via antioxidant buffering) may rapidly respond to oxidative challenges [45] and may surpass the rate of ROS production within mitochondria [121–123]. Such a powerful antioxidant mitochondrial capacity matches with the sustained ROS buffering capacity of mitochondria of remarkably long-lived species like the mole rat, where neither low mtROS production nor high non-mitochondrial antioxidant protections were described to be associated with their exceptional longevity [124]. In addition, in birds, mate choice is often based on sexually selected coloured signals caused by a class of antioxidant pigments known as carotenoids [125,126]. While it was traditionally suggested that carotenoids act as honest signals of individual quality through a trade-off mechanism—where their investment in ornamental coloration occurs at the expense of their systemic antioxidant role [127]—recent evidence proposes that the red coloration occurs rather via biochemical pathways that are intrinsically linked to mitochondrial efficiency [128]. For instance, coloration has been correlated in birds with liver mitochondrial coupling [54], and experimental studies in copepods have shown that mitochondrial uncoupling increases red pigment accumulation and metabolic rate [129].

The functional explanation for the positive effect of carotenoids on bioenergetics seems to be more related to an intimate structural interaction with the respiratory complexes [54] rather than to supposed antioxidant effects [130]. Similar complexity in the mechanisms linking coloration, mitochondrial functioning and immunity has been described [131]. However, one must keep in mind that considering mitochondrial antioxidant capacity or efficiency as mediators of life history trade-offs is not incompatible with the overall negative impact of cell ROS production on fitness, as proposed by the ‘oxidative stress theory’. The reduction of non-mitochondrial ROS production, such as the experimental inhibition of xanthine oxidase, which reduces the ageing rate (reviewed in [43]; see also [40]), is in line with such an idea. We propose that the impact of ROS needs to be understood within the context of mitohormesis (i.e. an adaptive response to moderate increases in mitochondrial ROS production [132]). Additionally, under stressful conditions, mitochondria may be unable to adapt to the new cell redox conditions and then tip into a pro-oxidant role [133].

### 2.5. The specific case of NADPH oxidases and its interaction with mitochondria

The ability of NOX to modulate cell redox status is likely not without consequences for an organism’s fitness. First, NOX4 has been shown to exert deleterious effects on respiratory chain function through an interaction with the electron transport complex I [134]. In addition, NOX enzymes actually oxidize the same reduced coenzymes used by the respiratory chain [135] and thus participate in the regulation of cell redox status and OxPhos efficiency. For instance, NOX4 interacts with the nuclear factor E2-related factor 2 (nrf2) signalling pathway, driving both NOX and mtROS productions, which contribute to the adaptive homeostatic response of cells [136]. When this response is impaired, it promotes ageing (see below).

Second, NOX is involved in the immune response and plays a role in human pathologies like chronic inflammation and cardiovascular diseases [113,137]. In addition, the expression of NOX1 is downregulated in king penguins (*Aptenodytes patagonicus*) when those marine birds acclimate to cold water at fledgling [138]. Together with other ROS producers that are jointly modulated by cold acclimation, this study suggests an implication of NOX-ROS-mediated signalling in physiological adaptations to environmental conditions. Another example lies in the involvement of NOX in insulin secretion [51], which opens the way for further interesting studies on animal adaptations to energy stress. In line with this, oxidative stress initiated by NOX2 can trigger the release of glucocorticoids (GCs), which, in turn, stimulate mitochondrial biogenesis, increase metabolism and oxygen consumption and thereby indirectly enhance ROS production [139]. Furthermore, GCs may exacerbate oxidative stress through non-metabolic effects by downregulating antioxidant defences [139]. The known cross-talk between NOX and the mitochondria [42,43] is also an interesting study subject for evolutionary biologists. Mitochondrial morphology and fusion–fission phenomena are mechanisms for regulating the efficiency of ATP production by mitochondria (reviewed in [30]). For example, the progressive increase in mitochondrial volume in red blood cells with age has been suggested as part of the process that mitigates the deleterious effect of age on ATP synthesis in zebra finches (*Taenopygia guttata*) [140]. ROS production by NOX is likely to be among the regulatory processes capable of altering mitochondrial morphology and function, providing an additional pathway for non-mitochondrial ROS modulation of mitochondrial bioenergetics [30].

## 3. General conclusion

The involvement of mitochondrial efficiency in ATP and ROS production is of increasing interest to the evolutionary biology community. This is driven by the near-universality of mitochondrial function across species (with Monocercomonoides as an exception [141]) and the dual role of mitochondria in producing both ATP and ROS. As such, mitochondria stand at the cross-roads of evolutionary trade-offs between life-history traits along energy and non-energy bases. The rapid advances in various animal models have nonetheless unravelled that mitochondrial bioenergetics actually modulate many more aspects of cell and organism homeostasis and physiology. In addition, the multiplicity of ROS producers within a cell and the heavy equipment of mitochondria with antioxidant enzymes raise an alternative view of the role played by mitochondria in trade-offs. Our understanding of the involvement of mitochondria as an ROS-buffering organelle and of non-mitochondrial ROS producers as modulators of trade-offs is in its infancy. We urgently need more descriptive studies on the relative importance of mitochondrial versus non-mitochondrial ROS production and its variability among species of different longevities or paces of life. Phylogenetic studies looking at the variability in DNA and protein sequences for enzymes involved in ROS production or antioxidant defences are likely to inform us about the specific evolution of given proteins having coevolved with particular adaptations or combinations of traits. Because natural selection operates at the individual level, variation in the activity of these enzymes among individuals in a given population and within individuals over time will also be highly informative in defining their fitness correlates. In addition, current topics of study, such as those dealing with animal responses to anthropogenic stressors and global change, would benefit from a better understanding of how mitochondrial antioxidant activity or extracellular ROS production (also implicated in detoxification) are modulated. Finally, extending the concept of mitohormesis may prove insightful, further investigating the largely neglected roles of mitochondrial/extra-mitochondrial ROS as cell signalling molecules involved in the definition of life history trade-offs.

## Data Availability

This article has no additional data.
